# Preventing confounding in observational studies in orthopedic trauma surgery through expert panels: a systematic review

**DOI:** 10.1007/s00068-024-02690-w

**Published:** 2025-01-24

**Authors:** Rolf H. H. Groenwold, L. X. van Rossenberg, D. P. J. Smeeing, R. M. Houwert, J. W. Schoones, S. P. J. Muijs, F. C. Oner, Y. de Jong, B. J. M. van de Wall

**Affiliations:** 1 Department of Clinical Epidemiology, Leiden University Medical Center, Albinusdreef 2, Leiden, 2333 ZA the Netherlands; 2 Department of Biomedical Data Sciences, Leiden University Medical Center, Leiden, the Netherlands; 3 Faculty of Health Sciences and Medicine, University of Lucerne, Lucerne, Switzerland; 4 Department of Trauma Surgery, Rijnstate Hospital, Arnhem, the Netherlands; 5 Department of Trauma Surgery, University Medical Center Utrecht, Utrecht, the Netherlands; 6 Directorate Research Policy, Leiden University Medical Center, Leiden, the Netherlands; 7 Department of Orthopedic Surgery, University Medical Center Utrecht, Utrecht, the Netherlands; 8 Acibadem International Medical Center, Amsterdam, the Netherlands; 9 Department of Thrombosis and Haemostasis, Leiden University Medical Center, Leiden, the Netherlands; 10 Department of Orthopedic and Trauma Surgery, Cantonal Hospital of Lucerne, Lucerne, Switzerland

**Keywords:** Confounding, Orthopedic trauma, Study design, Methodology, Natural experiment

## Abstract

**Purpose:**

Confounding in observational studies can be mitigated by selecting only those patients, in whom equipoise of both treatments is secured by experts’ disagreement over optimal therapy.

**Methods:**

We conducted a systematic review to identify observational studies in the field of orthopedic trauma surgery that utilized expert panels for patient inclusion in order to limit the potential for confounding.

**Results:**

Four studies were identified that used expert panels to select participants based on expert disagreement. Derived from these studies and our own experience, recommendations were made regarding reporting of the size and composition of the expert panel, the information the expert panel receives, criteria for disagreement, selection of patients, and statistical analysis.

**Conclusion:**

With this review we aim to provide insight into this study design and to stimulate discussions about the potential of expert panels to control for confounding in studies of medical treatments.

**Supplementary Information:**

The online version contains supplementary material available at 10.1007/s00068-024-02690-w.

## Introduction

Randomized trials are generally considered the gold standard to assess the effect of medical interventions, yet the design may not always be feasible or necessary. Particularly in case of treatment decisions that are largely based on physician and/or patients’ preferences, rather than patient characteristics, the potential for confounding may be limited and observational studies could provide valuable and credible information about treatment effects [1–4].

Even when the potential for confounding is deemed limited, additional steps could be taken to (further) reduce the potential for confounding. Commonly used approaches include regression-based correction for confounding factors and propensity score analysis [5, 6]. An alternative approach is to perform the observational study in a subset of patients who are very similar. Either, this selection could be based on a predefined set of characteristics (e.g., age, sex, comorbidities), or the selection could be at the discretion of an expert panel [3, 7]. In the latter case, confounding can be mitigated by selecting only those patients, in whom equipoise of both treatments is secured by experts’ disagreement over optimal therapy.

Examples of studies where expert panels are used to identify ‘similar’ patients in the field of orthopedic trauma surgery include studies on the operative versus non-operative treatment in traumatic thoracic and lumbar spinal fracture patients [8] and in proximal humerus fracture patients [9]. In these studies, it was hypothesized that disagreement between experts regarding the preferred treatment could identify a subset of patients for whom there appears to be ‘clinical equipoise’ and treatment groups are expected to be comparable.

It is unclear how often expert panels are used for patient selection in observational studies of medical treatments. Also, it is unknown which arguments are provided for choosing this design, how expert panels are set up, and what amount of information the experts are provided with. The aim of this paper is twofold. First, to report on a systematic review that aimed to identify observational studies that applied expert panels for patient inclusion in order to limit the potential for confounding, with a focus on orthopedic trauma surgery studies. Second, to discuss methodological challenges of this study design, based on findings in the literature and our own experience.

## Methods

The objective of this study was to (1) systematically review observational studies in the field of orthopedic trauma surgery that applied expert panels for patient inclusion in order to limit the potential for confounding; (2) provide insight in practical aspects of expert panels; and (3) provide insight in statistical analysis (notably confounding adjustment) of studies where expert panels were used. The protocol of the systematic review was registered online under Prospero ID 509,836 at https://www.crd.york.ac.uk/prospero/.

### Selection of studies

For a detailed description of the search strategy, we refer to the Appendix. Because we aimed to identify studies that applied a certain methodology, but not necessarily report it at such, we used a step-by-step method. First, based on four key publications that were already identified [7–10], keywords were extracted using Pubreminer, an online tool [https://hgserver2.amc.nl/cgi-bin/miner/miner2.cgi, last accessed 16 October 2024]. Second, a search string was created by an experienced medical librarian, using these keywords, synonyms and adjacency operators, which was then cross-checked against the key articles. Using this developed search string, PubMed, Embase, and WebOfScience were searched. Studies up until 13-09-2024 were included. Two researchers (RG & DS) independently performed title/abstract and full-text screening, as well as data extraction. Any disagreement was discussed with a third researcher (LvR).

Studies were considered eligible if they met the following criteria: (1) observational study in the field of orthopedic trauma surgery that applied expert panels for patient inclusion in order to limit the potential for confounding; (2) full-text article available; and (3) published in English. In addition, to limit the number of articles to be screened, the search was limited to (4) recent literature (articles published after 2000). Systematic reviews, meta-analyses, and conference abstracts were excluded. Studies in which an expert panel was not used as a means to reduce confounding, but only for a different reason (e.g., for outcome adjudication) were not included.

### Data extraction

A data extraction form was developed to extract information regarding the use of expert panels in observational orthopedic trauma studies, specifically the characteristics of such panels, how they are implemented, and whether statistical methods were used in addition to the expert panel to control for confounding. We only considered information provided in the article, in its appendices/ supplementary files, or if information regarding the panel was clearly linked to a reference. Extracted information included general information regarding the publication (first author, year and journal of publication, country of first author), information regarding study (patient group, treatment, comparator, main outcome, sample size), information regarding the expert panel (number of panel members, expertise of members, information that is provided to the panel, criteria to select patient into the study, number of patients in/excluded by panel, provided rationale), information about the potential for confounding among the study groups included by the expert panel (i.e., apparent comparability between treatment groups regarding prognostic characteristics), information regarding additional means to control for confounding, and information regarding consideration about (unmeasured) confounding.

### Analysis

Results are presented in a descriptive manner and no quantitative summaries are provided. No formal data synthesis was performed.

## Results

**Fig. 1:**
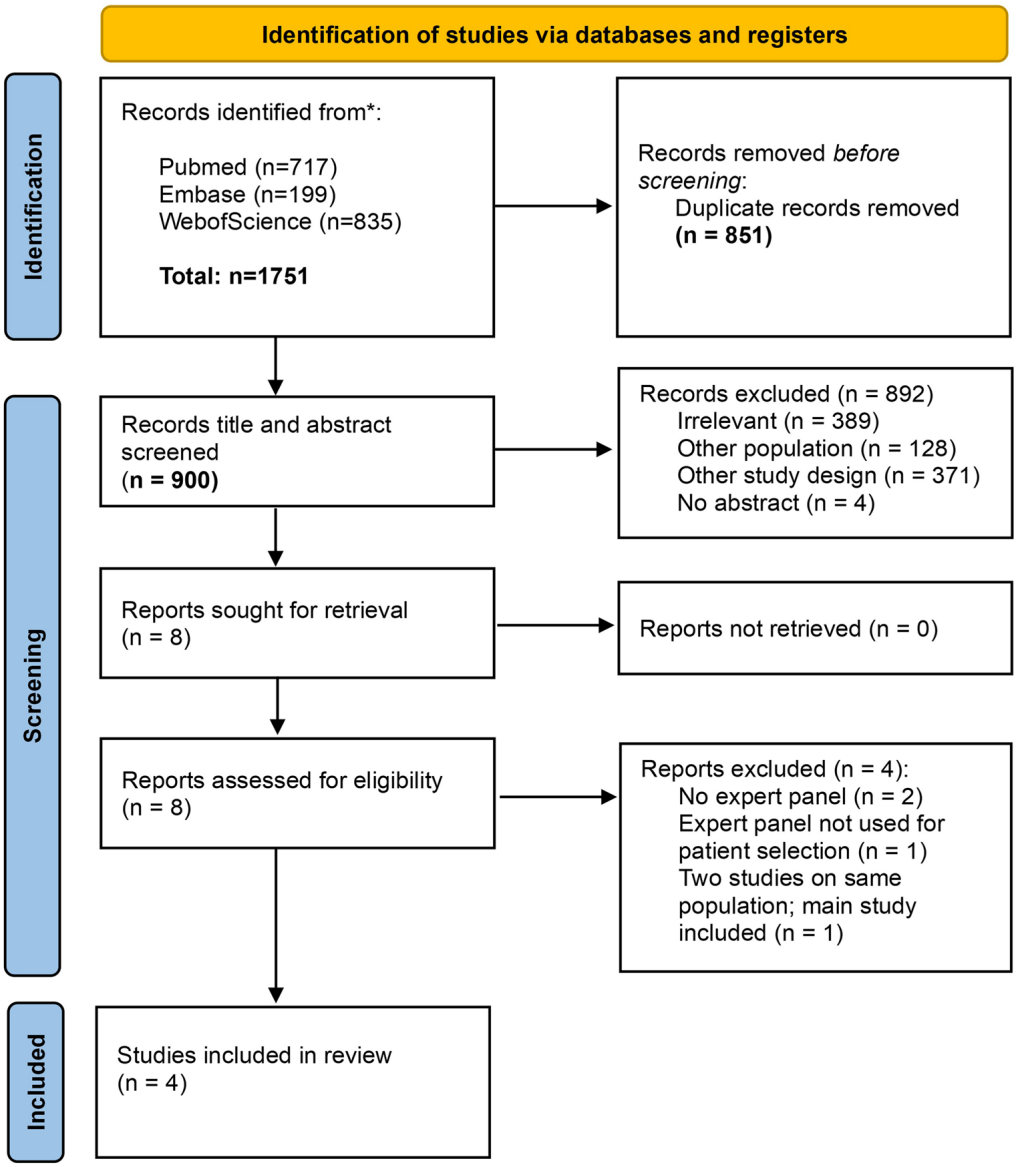
– Prisma flowchart of selection of observational studies in the field of orthopedic trauma surgery that utilized expert panels for patient inclusion

Figure 1 summarizes the literature search and article selection process. The literature search identified 900 articles after removal of duplicates. Of these, based on title and abstract screening, 389 articles were excluded because they were deemed irrelevant (e.g., qualitative study, consensus statement, cost-effectiveness analysis, or description of practice variation, single arm studies, imaging modality studies, or studies of fracture classification, studies of predictors of clinical outcomes) and 371 studies did not have the study design that we were interested in (e.g., randomized trials, observational studies without use of expert panel for selection of participants). Eight articles were included for full-text screening. After full-text screening, one article was excluded, because this study assessed surgeons’ preference, however without using it for patient selection [11]. Two other studies that were identified did not make use of an expert panel for selection, but were conducted as natural experiments that made use of practice variation between several hospitals [10, 12]. Two identified articles pertained to the same study [7, 8]. For one study [13], information was also obtained from a related paper [14]. Both reference and citation tracking of these eight articles did not result in any new potential eligible articles.

Four orthopedic trauma surgery studies that utilized expert panels for patient inclusion in order to limit the potential for confounding were selected for the review [8, 9, 13, 15]. Details of these studies are presented in Table 1. Two of the studies were ongoing at the time of this review, whereas one was completed and preliminary results for one were available. Three studies collected data prospectively, while one study used medical data collected in the past. All studies compared operative to nonoperative treatment and provided arguments for the choice of the study design. In all cases, the expert panel consisted of orthopedic (trauma) surgeons. In the study by Stadhouder et al. the panel consisted of two teams, whereas in the studies by Hoepelman et al. and by Van Veelen et al. the panels consisted of six individual surgeons and in the study described by Dandurand et al. the expert panel consisted of 22 spine trauma experts. In the study by Hoepelman et al., the definition of disagreement was based on incongruity between experts’ opinions in one country and actual treatment received in another country. In the studies by Van Veelen et al. and by Dandurand et al., it was simply defined based on disagreement within the expert panel. The article by Stadhouder et al. presented results of the study, including an assessment of the comparability between treatment groups, as well as a discussion on the potential of unmeasured confounding. The other articles described study protocols or preliminary results; hence, comparability between treatment groups could not be assessed yet. Furthermore, in those protocols no discussion of (the potential for) unmeasured confounding was included. None of the studies reported baseline characteristics of patients who were excluded by the expert panel.

**Table 1:** Characteristics of orthopedic trauma surgery studies that utilized expert panels for patient inclusion in order to limit the potential for confounding

	Stadhouder, 2008 [8]	Hoepelman, 2022 [9]	Dandurand, 2024 [13]	Van Veelen, 2024 [5]
***Study characteristics***				
*- p* *atient group*	Patients with traumatic thoracic and lumbar spine fractures	Patients with proximal humerus fracture	Patients with thoracolumbar burst fractures	Older patients with distal radius fracture
*- t* *reatment*	Operative treatment	Operative treatment	Operative treatment	Operative treatment
*- c* *omparator*	Nonoperative treatment	Nonoperative treatment	Nonoperative treatment	Nonoperative treatment
*- o* *utcome*	Neurologic improvement, pain, quality of life, and functional outcome	Functional outcome	Functional outcome (Oswestry Disability Index, ODI)	Functional outcome
*- s* *ample size*	2 × 95	Planned 220	Planned 208 subjects (137 operative and 71 nonoperative treatment)	Planned 2 × 46
***Expert panel***				
*- a* *rguments for the design*	“Surgeon equipoise is introduced as an inclusion criterion for a new blinded parallel group observational design. This has enabled the acquisition of balanced groups with unbiased evidence regarding the treatment outcomes of spine fractures.”	“By zooming in on a patient with a similar profile, confounding by patient characteristics will, to a large extent, be mitigated. One way of identifying patients with a similar profile is to identify those patients for whom experts disagree about the appropriate treatment.”	“Influencing the surgeons’ treatment decision is his/her background, education, experience, resources and the geographic area in which the surgeon trained and is practicing. “ “When the expert surgical community collectively has equipoise around treatment decisions, the influence of surgeon training, local experience, and resource availability likely all factor into the decision to recommend specific treatment much more than variables related to fracture morphology or classification.”	“In order to further minimize the influence of patient characteristics on treatment decisions, a selection could be made of those patients for whom a panel of physicians disagree regarding the preferred treatment option (ie, for whom there clearly is a clinical equipoise).”
*- number of members*	2 treatment teams (from the 2 participating university hospital centers)	“The expert panels will consist of an equal distribution of representatives from both countries, three orthopedic trauma surgeons from the participating Dutch centers and three orthopedic trauma surgeons from participating Swiss centers.”	22 experts	6 experts: “The expert panel will consist of 3 representatives from each “school.”[…] Half of them are generally in favor of conservative treatment (“school A”), while the others are generally in favor of operative treatment (“school B”).”
*- expertise*	Orthopedic surgeon	Orthopedic trauma surgeon	Spine trauma experts, “with demonstrated clinical and academic interest in spine trauma care. The expert panel included surgeons that were representative of a variety of geographic regions and reflected a heterogeneity in training backgrounds as well as patterns of clinical practice.”	Certified trauma surgeons
*- information provided to panel*	“The clinical and radiologic data of patients admitted in this period were blinded to the actual treatment they received and the clinical outcome”	“All relevant data to reach a “clinical” decision will be made available, including basic clinical information, radiographs, key images of CT-scans if available and radiology reports. The data will be presented to an expert panel of each country, blinded to the already initiated treatment.”	“…the radiographs of the 183 TL fracture records […] with all investigators and participants agnostic to the actual real-world treatment the patients received and also agnostic to the outcome results of the Spine TL A3/4 study itself”	“… anonymous radiographs including key images of computer tomography scans if available, and baseline characteristics including information regarding comorbidities and activity” “The panel will be blinded to the actual treatment received and the origin of the case.”
*- selection criteria*	Disagreement between the 2 treatment teams	“Patients can be included in the study if the majority of experts in one country, i.e., minimally two out of three, disagree with the received treatment in the other country”	“Equipoise threshold used was 77% (77:23 distribution of uncertainty or 17 vs. 5 experts).“	“Patients will [.] be included in the study if clinical equipoise is achieved, meaning 2 or more of the experts disagree with the rest of the panel.”
*- percentage selected*	30% (190/636)	Expected 40%	Preliminary results: 102/183 cases (55.7%) in the equipoise group	-
***Comparability***				
*- assessment of comparability of treatment groups*	Yes, by means of statistical tests comparing distributions of patient characteristics across treatment groups	-	-	-
*- adjustment for confounding*	Multivariable regression analysis, adjustment for 4 potential confounding variables: gender, polytrauma patients, neurologic impairment at admission, and the integrity of the posterior ligamentous complex	Propensity score matching, accounting for 5 potential confounding variables: age, sex, BMI, ASA score, and AO classification	-	Multivariable regression analysis, adjustment for 2 potential confounding variables: age and fracture type
*- considerations regarding unmeasured confounding*	“Because of the observational nature of the study, the most hazardous threat to the validity of the study remains confounding.” “An unavoidable limitation of the design is the inability to ensure that unknown and unmeasured prognostic factors are equally distributed between the treatment groups.”	-	-	-

## Discussion

### Main findings

This systematic review of observational studies in the field of orthopedic trauma surgery that used expert panels for patient inclusion based on disagreement regarding optimal treatment identified four studies. While this limited number of studies provides a (too) small basis to draw general conclusions about the potential of this study design in research practice, they illustrate how to implement the design, e.g., in terms of composition and size of the expert panel and the information that is provided to the panel.

The use of expert panels for patient selection based on disagreement has similarities with propensity score (PS) matching. In PS matching, treated subjects are matched with untreated subjects with a similar PS, provided that a potential match is available. As a result, the set of matched subjects mainly represents the patient subgroup that includes both treated and untreated patients, i.e. for whom there appears to be clinical equilibrium. An important difference is that in the case of PS matching, the PS has to be estimated from the data, according to a pre-specified model and based only on observed (confounding) variables [16]. When using an expert panel for patient selection, no model needs to be specified, but treatment decisions (and thus disagreement) can only be based on information provided to the experts. Both PS matching and patient selection based on expert disagreement are therefore potentially affected by unmeasured confounding. Because the two methods require different assumptions regarding confounding, their performance may differ depending on the context.

### Strengths and limitations

A strength of this study is that it is – to the best of our knowledge – the first review in the field of orthopedic trauma surgery to assess the use of expert panels to select participants based on disagreement. A limitation of our study is that we identified only four studies that used an expert panel that selects participants based on disagreement. This could be because the design is rarely applied, or because our literature search did not identify such studies (even though we modified our search to identify studies that applied a certain methodology, but not necessarily report it at such). We note that a strategy that aims to identify studies that applied a certain methodology based on title and abstract is prone to false-negative findings, because methodological aspects of a study are often not reported in the title or abstract of that study [17]. What is more, the design could be applied, yet if referred to using different terminology, we may have missed it. It therefore would be good to consider to develop reporting guidelines (e.g., additions to existing reporting guidelines such as STROBE [18] or RECORD [19]) that indicate how to report on studies that used an expert panel that selects participants based on disagreement. We acknowledge that some of the co-authors of this paper also co-authored one of the papers that was included in the review. This may have impacted our views on these papers. Finally, our focus on studies in the field of orthopedic trauma surgery may provide a limited view on medical research in general. While we are aware of one other medical study, which was in the field of orthodontics [3], that used an expert panel for patient inclusion based on disagreement, we do not know of other applications, which could provide useful experiences.

### Recommendations

Expert panels for patient selection based on disagreement have similarities to adjudication panels used in clinical trials. The latter consist of clinicians with sufficient experience and expertise, adjudication is blinded to treatment status and clinical outcomes, and adjudication is often performed independently [20]. An important difference, however, is that in the case of adjudication the aim is to minimize misclassification, whereas in the case of patient selection based on disagreement the aim is to identify the subgroup of patients for whom there appears to be a ‘clinical equipoise’.

Based on the findings in our review and our own experience with the design, we identified several additional issues that are important to consider when using an expert panel to select patients based on disagreement. Ideally, authors provide information on the six issues mentioned below, including the reasoning (arguments) behind the choices made.

1. **Panel composition.** A major issue is that of which experts are on the panel. Two experts with extreme, opposing, views may have disagreement for every patient, which would not reflect disagreement in clinical practice. Ideally, the expert panel is representative of the views among the larger group of those physicians who make treatment decision in clinical practice. In that case, selection into the study (because of disagreement by the expert panel) leads to a sample of patients for whom a larger group of physicians would have different viewpoints on the preferred treatment. Panels composed of experts from a single center may not reflect the diversity of viewpoints in the field.
2. **Panel size.** The number of panel members concerns the feasibility of the study as well as capturing the heterogeneity in views among experts in the field (where more members are likely to better reflect that heterogeneity). There could be a trade-off between the size of the panel and the number of patients selected, which also depends on criterion for disagreement (see below). If a patient is selected only in case of full disagreement (so that exactly half of the panel members recommend treatment A, whereas the other half recommends treatment B), disagreement is less likely in case of larger panels. The number of panel members need not correspond to the number of votes, provided that the experts that assess a particular case are representative of the group of experts (see composition of the panel).
3. **Disagreement.** What qualifies as disagreement among the panel members? If, for example, the panel consists of ten members, there clearly is disagreement if five physicians opt for one treatment and the other five physicians for the other treatment. However, what about four versus six, or one versus nine? More stringent criteria regarding disagreement could possibly limit potential confounding, yet also limit the precision of the results (due to smaller sample size) or feasibility (if still a certain number of patients needs to be recruited into the study). If sample size allows, researchers could test the impact of different selection criteria, e.g., by subgroup analysis of a particular combinations of disagreement (e.g., include five versus five votes only).
4. **Information.** What information is provided to the expert panel members? Preferably, the panel members receive all the information that they would have in practice to make their clinical judgment regarding the preferred treatment. Obviously, they should be blinded for the treatment that the patient actually received and blinded for the outcome the patients experienced (if already known). What is more, expert panel members should independently review each case, with no discussion among panel members, in order to better asses true disagreement.
5. **Selection.** In- and exclusion criteria of a study - such as having a particular fracture, being 18 years or older, and being capable of understanding the informed consent procedure - impact generalizability of the results of that study. Using an expert panel for additional selection of patients into the study further affects generalizability. The study by Stadhouder et al. nicely shows that the expert panels disagreed on the preferred treatment in approximately 30% of eligible patients. Results of the study apply to those patients who are like the 30% of patients that were included in the study. For readers to assess the generalizability of results, researchers should not only report characteristics of those included in the study, but also of those excluded by the expert panel (because there was agreement regarding the preferred treatment). This would facilitate an assessment of the generalizability of results by readers.
6. **Statistical analysis.** Selecting only those patients, in whom equipoise of both treatments is secured by experts’ disagreement regarding optimal therapy, is expected to limit confounding. However, additional adjustment for observed confounders may be necessary, in which case the statistical analysis should be described in detail. The assumption of no unmeasured confounding should be discussed, or – if this assumption is likely to be violated – the possible impact of unmeasured confounding needs attention, both in terms of direction and magnitude, for example through quantitative bias analysis.

## Conclusion

In this systematic review of studies in the field of orthopedic trauma surgery that used an expert panel to select participants based on disagreement only four studies were identified. This likely reflects unfamiliarity with the design. We hope this paper will stimulate discussions about the potential of this study design to control for confounding in studies of medical treatments and to increase its use and hence provide experience with and insight in its (methodological) challenges.

## Electronic supplementary material

Below is the link to the electronic supplementary material.

Supplementary Material 1

## Data Availability

No datasets were generated or analysed during the current study.

## References

[1] van de Wall BJM, Stadhouder A, Houwert RM, Oner FC, Beeres FJP, Groenwold RHH, NEXT Study Group. Natural experiments for orthopaedic trauma research: an introduction. Injury. 2023;54(2):429–34. 36402587 10.1016/j.injury.2022.11.028

[2] Houwert RM, Beks RB, Dijkgraaf MGW, Roes KCB, Öner FC, Hietbrink F, Leenen LPH, Groenwold RHH. Study methodology in trauma care: towards question-based study designs. Eur J Trauma Emerg Surg. 2021;47(2):479–84. 31664467 10.1007/s00068-019-01248-5 PMC8016800

[3] Korn EL, Baumrind S. Clinician preferences and the estimation of causal treatment differences. Stat Sci. 1998;13(3):209–35.

[4] Dvorak MF, Öner CF, Schnake K, Dandurand C, Muijs S. From Radiographic evaluation to treatment decisions in neurologically intact patients with thoraco-lumbar Burst fractures. Global Spine J. 2024;14(1suppl):S4–7. 10.1177/21925682231216584 PMC10867528 37991870

[5] Pouwels KB, Widyakusuma NN, Groenwold RH, Hak E. Quality of reporting of confounding remained suboptimal after the STROBE guideline. J Clin Epidemiol. 2016;69:217–24. 26327488 10.1016/j.jclinepi.2015.08.009

[6] Groenwold RHH, Dekkers OM, Methodology For the endocrinologist.: Basic aspects of confounding adjustment. Eur J Endocrinol. 2020;182(5):E5-E7. 10.1530/EJE-20-0075. PMID: 32069219. 10.1530/EJE-20-0075 32069219

[7] Stadhouder A, Oner FC, Wilson KW, Vaccaro AR, Williamson OD, Verbout AJ, Verhaar JA, de Klerk LW, Buskens E. Surgeon equipoise as an inclusion criterion for the evaluation of nonoperative versus operative treatment of thoracolumbar spinal injuries. Spine J. 2008;8(6):975–81. 18261964 10.1016/j.spinee.2007.11.008

[8] Stadhouder A, Buskens E, de Klerk LW, Verhaar JA, Dhert WA, Verbout AJ, Vaccaro AR, Oner FC. Traumatic thoracic and lumbar spinal fractures: operative or nonoperative treatment: comparison of two treatment strategies by means of surgeon equipoise. Spine. 2008;33(9):1006–17. 18427323 10.1097/BRS.0b013e31816c8b32

[9] Hoepelman RJ, Ochen Y, Beeres FJP, Frima H, Sommer C, Michelitsch C, Babst R, Buenter IR, van der Velde D, Verleisdonk EMM, Groenwold RHH, Houwert RM, van Heijl M. Let’s agree to disagree on Operative versus Nonoperative (LADON) treatment for proximal humerus fractures: study protocol for an international multicenter prospective cohort study. PLoS ONE. 2022;17(2):e0264477. 35213647 10.1371/journal.pone.0264477 PMC8880817

[10] Lecoultre Y, van de Wall BJM, Diwersi N, Pfarr SW, Galliker B, Babst R, Link BC, Beeres FJP. A natural experiment study: low-profile double plating versus single plating techniques in midshaft clavicle fractures-study protocol. PLoS ONE. 2023;18(9):e0291238. 37683048 10.1371/journal.pone.0291238 PMC10490911

[11] Mellema JJ, Janssen S, Schouten T, Haverkamp D, van den Bekerom MPJ, Ring D, Doornberg JN, Science of Variation Group. Intramedullary nailing versus sliding hip screw for A1 and A2 trochanteric hip fractures. Bone Joint J. 2021;103–B(4):775–81. 33591214 10.1302/0301-620X.103B.BJJ-2020-1490.R1

[12] Hoepelman RJ, Beeres FJP, Beks RB, Sweet AAR, Ijpma FF, Lansink KWW, van Wageningen B, Tromp TN, Link BC, van Veelen NM, Hoogendoorn JM, de Jong MB, van Baal MCP, Leenen LPH, Groenwold RHH, Houwert RM. Non-operative vs. operative treatment for multiple rib fractures after blunt thoracic trauma: a multicenter prospective cohort study. Eur J Trauma Emerg Surg. 2023;49(1):461–71. 36008560 10.1007/s00068-022-02093-9 PMC9925506

[13] Dandurand C, Dvorak MF, Hazenbiller O, Bransford RJ, Schnake KJ, Vaccaro AR, Benneker LM, Vialle E, Schroeder GD, Rajasekaran S, El-Skarkawi M, Kanna RM, Aly MM, Holas M, Canseco JA, Muijs S, Popescu EC, Tee JW, Camino-Willhuber G, Joaquim AF, Keynan O, Chhabra HS, Bigdon S, Spiegel U, Öner CF. Using equipoise to determine the radiographic characteristics leading to Agreement on best treatment for Thoracolumbar Burst fractures without neurologic deficits. Global Spine J. 2024;14(1suppl):S25–31. 10.1177/21925682231215770 PMC10867529 38324599

[14] Dandurand C, Öner CF, Hazenbiller O, Bransford RJ, Schnake K, Vaccaro AR, Benneker LM, Vialle E, Schroeder GD, Rajasekaran S, El-Skarkawi M, Kanna RM, Aly M, Holas M, Canseco JA, Muijs S, Popescu EC, Tee JW, Camino-Willhuber G, Joaquim AF, Keynan O, Chhabra HS, Bigdon S, Spiegel U, Dvorak MF. Understanding decision making as it influences treatment in Thoracolumbar Burst fractures without neurological deficit: conceptual Framework and Methodology. Global Spine J. 2024;14(1suppl):S8–16. 10.1177/21925682231210183 PMC10867530 38324598

[15] van Veelen NM, van de Wall BJM, Hoepelman RJ, IJpma FFA, Link BC, Babst R, Groenwold RHH, van der Velde D, Diwersi N, van Heijl M, Houwert RM, Beeres FJP. Let’s agree to disagree on Operative Versus Nonoperative Treatment for Distal Radius fractures in older people: protocol for a prospective International Multicenter Cohort Study. JMIR Res Protoc. 2024;13:e52917. 38349719 10.2196/52917 PMC10900084

[16] Groenwold RHH, Dekkers OM, le Cessie S. Ten things to remember about propensity scores. Eur J Endocrinol. 2024;191(1):E1–4. 38872400 10.1093/ejendo/lvae067

[17] Penning de Vries BBL, van Smeden M, Rosendaal FR, Groenwold RHH. Title, abstract, and keyword searching resulted in poor recovery of articles in systematic reviews of epidemiologic practice. J Clin Epidemiol. 2020;121:55–61. 31982541 10.1016/j.jclinepi.2020.01.009

[18] Vandenbroucke JP, von Elm E, Altman DG, Gøtzsche PC, Mulrow CD, Pocock SJ, Poole C, Schlesselman JJ, Egger M, STROBE Initiative. Strengthening the reporting of Observational studies in Epidemiology (STROBE): explanation and elaboration. Int J Surg. 2014;12(12):1500–24. 25046751 10.1016/j.ijsu.2014.07.014

[19] Benchimol EI, Smeeth L, Guttmann A, Harron K, Moher D, Petersen I, Sørensen HT, von Elm E, Langan SM, RECORD Working Committee. The REporting of studies conducted using Observational routinely-collected health data (RECORD) Statement. PLoS Med. 2015;12(10):e1001885. 26440803 10.1371/journal.pmed.1001885 PMC4595218

[20] Kahan BC, Feagan B, Jairath V. A comparison of approaches for adjudicating outcomes in clinical trials. Trials. 2017;18:266. 28595589 10.1186/s13063-017-1995-3 PMC5465459

