# Supplementary material for: Preventing confounding in observational studies in orthopedic trauma surgery through expert panels: a systematic review
Source: Eur J Trauma Emerg Surg. 2025 Jan 24;51(1):36. doi: 10.1007/s00068-024-02690-w (PMC11762208; doi:10.1007/s00068-024-02690-w)
Supplement: Supplementary file 1 — Supplementary Material 1 [file 68_2024_2690_MOESM1_ESM.docx]

**Appendix: expanded search methods**

A PubMed search string was crafted in collaboration with an experienced medical librarian and validated against four previously published studies employing similar methodologies:

- Hoepelman *et al.*, 2022, PMID 35213647
- Stadhouder *et al.*, 2008, PMID 18261964
- Stadhouder *et al.*, 2008, PMID 18427323
- Lecoultre *et al.*, 2023, PMID 37683048

Utilizing PubMed PubReMiner (https://hgserver2.amc.nl/cgi-bin/miner/miner2.cgi), the common terms from these publications were extracted and expanded upon, resulting in the inclusion of search terms such as equipoise (Therapeutic Equipoise, surgeon equipoise), comparison (comparison treatment, compare treatment, etc.), expert panel, and natural experiment. Adjacency operators were then applied to compose the final search string.

(("Therapeutic Equipoise"[Mesh] OR "surgeon equipoise"[tw] OR "equipoise"[tw] OR "comparison treatment"[title/abstract:~3] OR "comparison treatments"[title/abstract:~3] OR "compare treatment"[title/abstract:~3] OR "compare treatments"[title/abstract:~3] OR "treatment variation"[tw] OR "treatment variation"[title/abstract:~6] OR "treatments variation"[title/abstract:~6] OR "expert panels"[tw] OR "expert panel"[tw] OR "natural experiments"[tw] OR "natural experiment"[tw] OR "expert panels"[title/abstract:~3] OR "expert panel"[title/abstract:~3])

Our focus being on the utilization of expert panels within the domain of orthopaedic surgery, our search was narrowed to fractures using the following terms:

("Fractures, Bone"[mesh] OR "fracture"[tw] OR "fractures"[tw] OR "fractur*"[tw] OR "Bone and Bones/injuries"[Mesh])

Subsequently, we implemented a filter to exclude case reports and various types of reviews (including narrative reviews, systematic reviews, and/or meta-analyses) from our search. During a preliminary search, a substantial number of studies focusing on the development or evaluation of guidelines within our field of interest were identified. Given that a predominant approach in these studies was the Delphi method, we specifically applied a filter to exclude studies utilizing this method as well.

(("Case Reports"[ptyp] OR "case report"[ti] OR "case rep"[all fields] OR "Review"[ptyp] OR "review"[ti] OR "systematic"[sb] OR "meta-analysis"[pt] OR "metaanal*"[ti] OR "meta anal*"[ti] OR "Practice Guideline"[pt] OR "guideline"[ti] OR "guidelines"[ti]) NOT ("Clinical Study"[ptyp] OR "trial"[ti] OR "RCT"[ti])) AND (english[la] OR dutch[la]) AND ("2000/01/01"[PDAT] : "3000/12/31"[PDAT]) NOT ("Delphi Technique"[mesh] OR "delphi"[tw]) NOT ("Animals"[mesh] NOT "Humans"[mesh]))

This resulted in the final search string for PubMed, which yielded 717 titles on 13-09-2024.

(("Therapeutic Equipoise"[Mesh] OR "surgeon equipoise"[tw] OR "equipoise"[tw] OR "comparison treatment"[title/abstract:~3] OR "comparison treatments"[title/abstract:~3] OR "compare treatment"[title/abstract:~3] OR "compare treatments"[title/abstract:~3] OR "treatment variation"[tw] OR "treatment variation"[title/abstract:~6] OR "treatments variation"[title/abstract:~6] OR "expert panels"[tw] OR "expert panel"[tw] OR "natural experiments"[tw] OR "natural experiment"[tw] OR "expert panels"[title/abstract:~3] OR "expert panel"[title/abstract:~3]) AND ("Fractures, Bone"[mesh] OR "fracture"[tw] OR "fractures"[tw] OR "fractur*"[tw] OR "Bone and Bones/injuries"[Mesh]) NOT (("Case Reports"[ptyp] OR "case report"[ti] OR "case rep"[all fields] OR "Review"[ptyp] OR "review"[ti] OR "systematic"[sb] OR "meta-analysis"[pt] OR "metaanal*"[ti] OR "meta anal*"[ti] OR "Practice Guideline"[pt] OR "guideline"[ti] OR "guidelines"[ti]) NOT ("Clinical Study"[ptyp] OR "trial"[ti] OR "RCT"[ti])) AND (english[la] OR dutch[la]) AND ("2000/01/01"[PDAT] : "3000/12/31"[PDAT]) NOT ("Delphi Technique"[mesh] OR "delphi"[tw]) NOT ("Animals"[mesh] NOT "Humans"[mesh]))

The search was then rewritten and applied to Web of Science (n=835).

((TI=("surgeon equipoise" OR "equipoise" OR (("comparison"NEAR/3 "treatment") OR ("comparison"NEAR/3 "treatments") OR ("compare"NEAR/3 "treatment") OR ("compare"NEAR/3 "treatments")) OR "treatment variation" OR (("treatment"NEAR/6 "variation") OR ("treatments"NEAR/6 "variation")) OR "expert panels" OR "expert panel" OR "natural experiments" OR "natural experiment" OR (("expert"NEAR/3 "panels") OR ("expert"NEAR/3 "panel"))) OR AK=("surgeon equipoise" OR "equipoise" OR (("comparison"NEAR/3 "treatment") OR ("comparison"NEAR/3 "treatments") OR ("compare"NEAR/3 "treatment") OR ("compare"NEAR/3 "treatments")) OR "treatment variation" OR (("treatment"NEAR/6 "variation") OR ("treatments"NEAR/6 "variation")) OR "expert panels" OR "expert panel" OR "natural experiments" OR "natural experiment" OR (("expert"NEAR/3 "panels") OR ("expert"NEAR/3 "panel"))) OR AB=("surgeon equipoise" OR "equipoise" OR (("comparison"NEAR/3 "treatment") OR ("comparison"NEAR/3 "treatments") OR ("compare"NEAR/3 "treatment") OR ("compare"NEAR/3 "treatments")) OR "treatment variation" OR (("treatment"NEAR/6 "variation") OR ("treatments"NEAR/6 "variation")) OR "expert panels" OR "expert panel" OR "natural experiments" OR "natural experiment" OR (("expert"NEAR/3 "panels") OR ("expert"NEAR/3 "panel")))) AND (TI=("Fractures" OR "fracture" OR "fractur*") OR AK=("Fractures" OR "fracture" OR "fractur*") OR AB=("Fractures" OR "fracture" OR "fractur*")) NOT (TI=("Case Report" OR "review" OR "meta analysis" OR "metaanal*" OR "meta anal*" OR "Guideline*") OR AK=("Case Report" OR "review" OR "meta analysis" OR "metaanal*" OR "meta anal*" OR "Guideline*") OR DT=("review")) AND LA=(english OR dutch) AND PY=(2000 OR 2001 OR 2002 OR 2003 OR 2004 OR 2005 OR 2006 OR 2007 OR 2008 OR 2009 OR 2010 OR 2011 OR 2012 OR 2013 OR 2014 OR 2015 OR 2016 OR 2017 OR 2018 OR 2019 OR 2020 OR 2021 OR 2022 OR 2023 OR 2024) NOT TS=("delphi") NOT TI=("veterinary" OR "rabbit" OR "rabbits" OR "animal" OR "animals" OR "mouse" OR "mice" OR "rodent" OR "rodents" OR "rat" OR "rats" OR "pig" OR "pigs" OR "porcine" OR "horse" OR "horses" OR "equine" OR "cow" OR "cows" OR "bovine" OR "goat" OR "goats" OR "sheep" OR "ovine" OR "canine" OR "dog" OR "dogs" OR "feline" OR "cat" OR "cats") NOT DT=("meeting abstract"))

… and Embase (n=199).

(("Equipoise"/ OR "surgeon equipoise".mp OR "equipoise".mp OR (("comparison"ADJ3 "treatment") OR ("comparison"ADJ3 "treatments") OR ("compare"ADJ3 "treatment") OR ("compare"ADJ3 "treatments")).ti,ab OR "treatment variation".mp OR (("treatment"ADJ6 "variation") OR ("treatments"ADJ6 "variation")).ti,ab OR "expert panels".mp OR "expert panel".mp OR "natural experiments".mp OR "natural experiment".mp OR (("expert"ADJ3 "panels") OR ("expert"ADJ3 "panel")).ti,ab) AND (exp "Fractures"/ OR "fracture".mp OR "fractures".mp OR "fractur*".mp) NOT (("Case Report"/ OR "case report".ti OR (case AND (report OR reports)).jw OR "Review"/ OR "review".ti OR "systematic review"/ OR "meta analysis"/ OR "metaanal*".ti OR "meta anal*".ti OR exp "Practice Guideline"/ OR "guideline".ti OR "guidelines".ti) NOT ("Clinical Study"/ OR "trial".ti OR "RCT".ti)) AND (english.la OR dutch.la) AND 2000:2024.(sa_year) NOT ("Delphi Study"/ OR "delphi".mp) NOT (exp "Animals"/ NOT exp "Humans"/) NOT (conference review or conference abstract).pt)
